# Technology-based applications for relatives and caregivers of people with advanced breast cancer—a scoping review

**DOI:** 10.1007/s00520-026-10712-z

**Published:** 2026-05-05

**Authors:** Sara Marquard, Isabel Jalaß, Manfred Hülsken-Giesler

**Affiliations:** 1 Faculty of Business Management and Social Sciences, Department of Nursing Science, Osnabrück University of Applied Sciences, Osnabrück, Germany; 2 Department of Nursing Science, Institute of Health Research and Education, Osnabrück University, Osnabrück, Germany

**Keywords:** Breast cancer, Advanced cancer, Informal caregivers, Digital health, Mobile applications, Psychological well-being, Quality of life

## Abstract

**Objective:**

To provide an overview of the current state of international research on technology-based applications for informal caregivers of people with advanced cancer, with a particular focus on advanced breast cancer.

**Methods:**

This scoping review was conducted in accordance with the Joanna Briggs Institute methodology and reported in line with the PRISMA-ScR guideline. The PCC framework was used to define the search terms, and develop the search strategy for the databases PubMed, CINAHL, and Web of Science. The systematic search identified 13 relevant articles describing ten different technology-based applications. One additional article was identified through a manual search.

**Results:**

In total, 14 studies covering ten distinct interventions were included. Some interventions were adapted from face-to-face programmes for digital delivery, whereas only a minority were explicitly informed by theoretical models from psychology or health science. The included studies addressed five main areas: informational support, mental and psychosocial support and enhancement of quality of life, physical and practical support, communication support, and preparation for caregiving and death. Evaluations reported predominantly positive findings, particularly with regard to quality of life, anxiety, depressive symptoms, and coping. However, most studies focused on advanced cancer more broadly rather than on advanced breast cancer specifically.

**Conclusions:**

The reviewed literature suggests that technology-based interventions for informal caregivers of people with advanced cancer are available in several countries and address a range of support needs. However, no intervention tailored to relatives of patients with advanced breast cancer was identified as having been fully developed and evaluated. The findings highlight the need for future research on targeted, sustainable digital support for this group. The development of the Gesi-BK platform is based on the results of this scoping review.

**Supplementary information:**

The online version contains supplementary material available at 10.1007/s00520-026-10712-z.

## Background

One in five people worldwide will develop cancer during their lifetime. In 2022, over 19 million people worldwide were living with cancer, and approximately 9.7 million people died from the disease. Breast cancer was the most commonly diagnosed cancer type globally, with more than 2.29 million new cases [1]. In Germany, approximately 4.5 million people currently have or have had cancer [2]. Breast cancer is one of the most prevalent cancer types in Germany. In 2023, 75,090 women and 770 men were diagnosed with the disease [3, 4]. Advanced breast cancer is incurable [5]. A patient’s diagnosis has an impact that extends beyond the individual, affecting family members and significant others. This group is heterogeneous and includes partners, children, friends, employed individuals, and older adults living in a wide range of circumstances and fulfilling different roles within the caregiving context [6–8]. Given the variability in disease progression, care and nursing interventions also differ considerably. Dynamic disease trajectories and the associated shifting demands placed on relatives and family members are characteristic [9, 10]. Relatives and family members, the target group of this review, are therefore exposed to multiple stressors and represent a particularly vulnerable group, often experiencing substantial distress and worry [11–13]. This situation can give rise to numerous challenges, including emotional, organisational, and time-related difficulties. Consequently, conflicts may arise between caregiving responsibilities and other obligations. Against this background, it is essential for healthcare systems to implement structured measures to ensure that caregivers receive adequate psychosocial support and professional care [13]. In this context, advances in digital technology in healthcare are of particular importance, particularly because their development and implementation were accelerated by the COVID-19 pandemic [14, 15].

Recent research has shown that digital support for informal caregivers can improve mental well-being, strengthen self-efficacy, enhance caregiving competencies, increase overall quality of life, and reinforce social support networks and coping abilities [16, 17]. At present, however, the number of available applications is limited, and the extent of their evaluation remains modest [18]. More mobile applications should be developed and evaluated in order to provide services tailored to specific target groups [17, 19].

The present review originated within the project Caring Together for Advanced Breast Cancer (Gesi-BK; 2024-2026), funded by the German Federal Ministry of Research, Technology and Space (BMFTR). The project aims to develop a digital application to provide information, support, and networking opportunities for family members and informal caregivers of people with advanced breast cancer. Against this backdrop, the present scoping review identifies and maps relevant technology-based applications and, by synthesising the available evidence, seeks to clarify the current state of research, identify gaps in existing support provision, and provide a foundation for the future development and evaluation of technology-based caregiver support.

## Methods

A scoping review approach was selected to provide a comprehensive overview of current international publications by including all types of evidence [20, 21].

The review was conducted in accordance with the Joanna Briggs Institute (JBI) methodology [20]. The literature search was carried out from November to December 2025 and updated in March 2026. Reporting followed the Preferred Reporting Items for Systematic Reviews and Meta-Analysis extension for Scoping Reviews (PRISMA-ScR) guidelines (see Appendix 1) [21].

### Search strategy

At the outset of the study, a PCC framework was developed to define the research question and search strategy [20]:

- P (Population) = caregivers, relatives
- C (Concept) = technology-based interventions
- C (Context) = advanced breast cancer/palliative care/oncology

The research question guiding this study was: “What technology-based applications are currently available for informal caregivers and relatives of individuals with advanced cancer, particularly breast cancer?”

### Study selection

The review focused on adult friends, partners and family members acting as informal caregivers of people with advanced cancer, explicitly excluding professional caregivers and children. The inclusion criteria were intentionally broad. Articles were eligible if they addressed digital support services of any kind within the context of (advanced) cancer, particularly advanced breast cancer. This review included only articles published in English or German. Articles focusing on chronic diseases or survivorship were excluded. Literature published before 2012 was also excluded, as the review aimed to reflect the current state of technology and recent developments (see Table 1).

**Table 1:** Inclusion and exclusion criteria

	Inclusion criteria	Exclusion criteria
Population	Friends and family (informal caregivers), adults	Professional caregivers, children
Concept	Technology-based support interventions in any form (apps, websites, forums)	Face-to-face interventions, telephone interventions
Context	Advanced (breast) cancer/palliative care	Other chronic diseases, survivorship
Language	English or German	All other languages
Type of literature	All	None
Publication date	2012–2025	Literature published before 2012

The search strings were developed on the basis of the PCC framework and the above inclusion and exclusion criteria. The search was conducted in PubMed, CINAHL, and Web of Science. During the revision process, the search strategy was updated include mobile health-specific terminology (e.g. Mobile Applications and related keywords, such as “smartphone*”, and “app-based”) in order to ensure comprehensive identification of app-based interventions. The updated search yielded additional records; however, none met the inclusion criteria. Table 2 presents the full search strings used for the different databases.

**Table 2:** Search strategies

Database	Search string
PubMed	(“Internet”[MeSH] OR “Telemedicine”[MeSH] OR “Mobile Applications”[MeSH] OR “online”[Title/Abstract] OR “web-based”[Title/Abstract] OR “internet-based”[Title/Abstract] OR “digital”[Title/Abstract] OR “eHealth”[Title/Abstract]OR “mHealth”[Title/Abstract] OR “mobile health”[Title/Abstract] OR “mobile application*”[Title/Abstract] OR “mobile app*”[Title/Abstract] OR “smartphone*”[Title/Abstract] OR “app-based”[Title/Abstract]) AND (“Caregivers”[MeSH] OR “caregivers”[Title/Abstract] OR “family”[Title/Abstract] OR “relatives”[Title/Abstract] OR “informal carer*”[Title/Abstract]) AND (“Breast Neoplasms”[MeSH] OR “breast cancer”[Title/Abstract] OR “advanced cancer”[Title/Abstract] OR “metastatic cancer”[Title/Abstract])
CINAHL	(MH “Internet” OR MH “Telemedicine” OR MH “Mobile Applications”OR TI online OR AB online OR TI “web-based” OR AB “web-based” OR TI “internet-based” OR AB “internet-based” OR TI digital OR AB digital OR TI eHealth OR AB eHealthOR TI mHealth OR AB mHealthOR TI “mobile health” OR AB “mobile health” OR TI “mobile app*” OR AB “mobile app*” OR TI “mobile application*” OR AB “mobile application*” OR TI smartphone* OR AB smartphone* OR TI “app-based” OR AB “app-based”) AND (MH “Caregivers” OR TI caregiver* OR AB caregiver* OR TI “informal carer*” OR AB “informal carer*” OR TI family OR AB family OR TI relative* OR AB relative*) AND (MH “Breast Neoplasms” OR TI “breast cancer” OR AB “breast cancer” OR TI “advanced cancer” OR AB “advanced cancer” OR TI “metastatic cancer” OR AB “metastatic cancer”) NOT (TI patient* OR AB patient* OR TI “self-management” OR AB “self-management”)
Web of Science	TS = ((online OR “web-based” OR “internet-based” OR digital OR eHealth OR telemedicine OR mHealth OR “mobile health” OR “mobile app*” OR “mobile application*” OR smartphone* OR “app-based”) AND (caregiver* OR “informal carer*” OR family OR relative*) AND (“breast cancer” OR “advanced cancer” OR “metastatic cancer”) ) NOT TS = (“self-management” OR patient*)

## Results

### Included studies

**Fig. 1:**
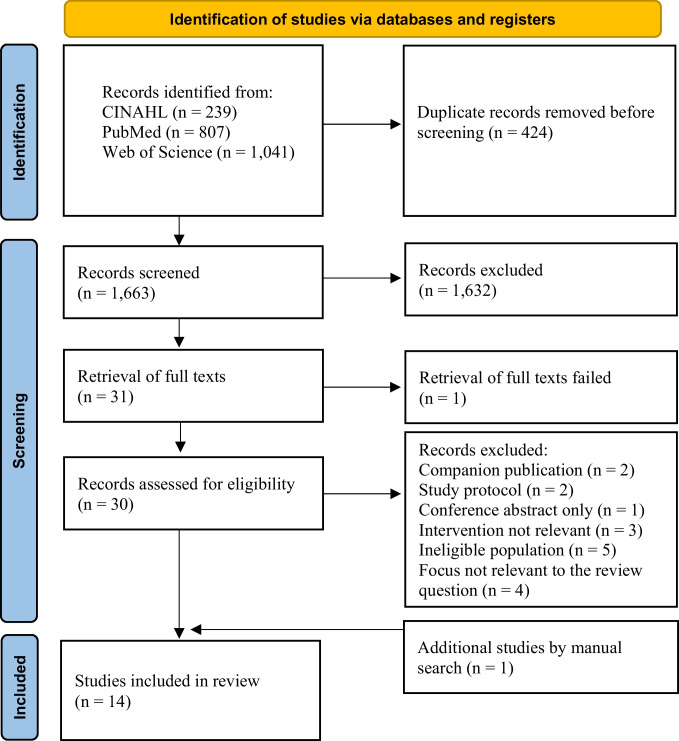
PRISMA flow diagram

The search results from all three databases were imported into Rayyan-software, an online tool for review management that automatically identifies and removes duplicate records. After deduplication, two authors (SM and IJ) independently screened the records. Thirty articles underwent full-text screening, of which 13 were eligible for inclusion in the review. Inclusion required reviewer consensus which was reached through discussion. In addition to the systematic database search, a manual search was conducted, which resulted in the inclusion of 1 additional article. In total, 14 studies were included in this review. The corresponding PRISMA flow diagram [22] is shown in Fig. 1.

### Study context

Data were extracted from the included studies and categorised according to several criteria (Table 3).

**Table 3:** Summary of the included studies

	*n*	%
Publication year		
2013–2017	3	21
2018–2021	5	36
2022–2025	6	43
Country/region		
Australia	2	14
Europe	2	14
Japan	1	7
Singapore	2	14
UK	1	7
USA	6	43
Methods		
Semi-structured qualitative interviews	3	21
Mixed methods	3	21
RCT	8	57
Sample		
Patients and informal caregivers	5	36
Only informal caregivers	9	64
Cancer type		
Only breast cancer	2	14
All types of cancer	10	71
Not specified	2	14
Disease stage		
Advanced cancer only	12	86

The included studies were conducted in Australia [23, 24], Europe [25, 26], Japan [27], Singapore [28, 29], the UK [30], and the USA [31–36]. Most studies were published between 2022 and 2025.

Three studies used a qualitative methodology [23, 26, 28], eight used a quantitative approach [25, 27, 29, 30, 33–36], and three employed a mixed-methods design [24, 31, 32].

Two studies describing the same technology focus exclusively on breast cancer [23, 24]. A further eight not only included a substantial proportion of relatives of patients with breast cancer, but also considered other cancers [25–27, 29, 30, 32–34]. In two studies relating to the same intervention, no relatives of individuals with breast cancer were included in the study population; however, the intervention was designed generically for relatives of patients with advanced cancer [31, 36]. In two additional studies, no information was provided on the type of cancer represented in the study population [28, 35].

Twelve studies focused exclusively on advanced cancer [25–36].

### Types of technology-based interventions

Across the 14 included studies, ten distinct applications were identified. The individual applications and their associated studies are described below. An overview of all included studies is provided in Appendix 2.

The studies by Bamgboje-Avodele et al. [23] and Goria et al. [24] address the technology “Care Assist”, an online intervention intended for male relatives of women with breast cancer and currently still in the development stage. Accordingly, these studies focus on the needs of relatives, technological requirements, and relevant framework conditions.

Carr et al. [31] and Pensak et al. [36] examined the web-based application “Pep-Pal” for relatives of patients with advanced cancer. Pep-Pal is derived from an existing face-to-face intervention. The application comprises an introduction, nine full sessions, and brief supplementary “Mini-Peps” that provide instructions for activities. Its primary focus is psychoeducation. Pep-Pal can be accessed at any time via smartphone, tablet, or computer through a website. The evaluated outcomes included usability and perceived helpfulness [31], as well as anxiety, depression, stress, current health status, sexual function, and health [36].

The “Resilient Living Program” (RLP) was evaluated in the study by Chesak et al. [32]. RLP is a mindfulness-based intervention for relatives of patients with advanced cancer and comprises four individual sessions delivered via teleconsultation. Additional video modules can also be completed. The programme is based on Stress Management and Resiliency Training (SMART), adapted to the specific needs of the target group. The evaluation addressed anxiety, stress, quality of life, sleep, resilience, fatigue, and participants’ experiences with the programme.

The article by Chih et al. [33] presents and evaluates the web-based Comprehensive Health Enhancement Support System (CHESS). CHESS is used to regularly assess the current situation, mood, and stress levels of patients with advanced cancer and their relatives. Preparedness and caregiver burden were evaluated as outcomes.

The effectiveness of the Online Daily Diary (ONDIARY) was examined in the article by Ito and Tadaka [27]. This online diary is intended to reduce stress, maintain mental health, and improve the emotional competence of relatives of people with advanced cancer through daily entries. Quality of life and depression were assessed as outcomes.

Kubo et al. [34] investigated the effects of the commercially available interventions “Headspace” and “eMindful” on quality of life, mindfulness, and symptoms of stress, anxiety, and depression in patients with advanced cancer and their relatives. These applications are already available on the market, but were not originally developed specifically oncological settings.

The Caring for the Caregiver Program (CCP) was developed for relatives of people with advanced cancer and comprises a face-to-face session, video clips, telephone calls, and an online support group. The programme is based on Bandura’s self-efficacy theory [37] and aims to improve communication, provide information, and strengthen social support. The publications by Leow et al. [29] and Leow and Chan [28] describe the intervention and present evaluation findings. The outcomes examined included quality of life, social support, stress, depression, relationship quality, self-efficacy, benefits of caregiving, knowledge, resources, and preparation for death [28], as well as the participants’ perception of the intervention [29].

The Digital Supportive Care Awareness and Navigation (D-SCAN) app was developed to raise awareness of services and resources among patients with advanced cancer and their relatives. In the study by Merz et al. [30], the app was assessed in terms of feasibility, user-satisfaction, and effectiveness.

Parker Oliver et al. [35] examined the potential of digital support groups on Facebook for relatives of people with advanced cancer receiving hospice care. The intervention focused primarily on the provision of knowledge through videos addressing topics such as hospice care, pain management, decision-making, and the dying process. Additional materials and discussion posts were also provided within moderated groups. The specific aspects addressed varied according to the current needs of the group members at the time. Anxiety, health, quality of life, and stress were included as outcome measures in the evaluation.

The iFOCUS intervention programme is based on the face-to-face FOCUS intervention originally developed in the USA and later adapted for Europe (FOCUS+), before being transferred to a web-based format. iFOCUS comprises five components focusing on communication, resilience, coping, uncertainty, and symptom management. Relatives and patients affected by advanced cancer engage with the programme together. The intervention is based on Lazarus and Folkman’s transactional model of stress and coping [38] and on a salutogenic approach [38, 39]. Van Goethem et al. [26] describe the development process, whereas De Vlemnick et al. [25] report evaluation results, which included emotional functioning, self-efficacy, quality of life, perceived benefits, and coping as outcome measures.

### Content and impact of technology-based interventions

The technology-based interventions address a range of topics and support functions, which can be grouped into five categories: (1) informational support, (2) mental and psychosocial support and enhancement of quality of life, (3) physical and practical support, (4) communication support, and (5) preparation for caregiving and death.

#### Informational support

In the two articles describing the design of an application, the integration of educational and informational content is highlighted as an important component [23, 24].

The study by Bamgboje-Ayodele et al. [23] found that male relatives of women with breast cancer felt that they had not received sufficient information. They reported wanting to know what to expect and which questions to ask. They also indicated that an online intervention should include educational content on treatment options, side effects, and caregiving-related topics. In addition, the study identified a need for information on legal and organisational issues, such as reconciling caregiving with employment and accessing financial support. The Delphi study by Goria et al. [24] similarly identified the provision of basic information on breast cancer, treatment options, and coping strategies as an important element of Care Assist.

Seven of the included applications provide information to people with advanced cancer and their relatives or are explicitly designed to convey knowledge.

In the study of the web-based CHESS application, relatives documented their situation and received clinical support when needed. They could also use the application to note questions for their next appointment with clinicians. The technology is intended to facilitate the timely exchange of information between relatives and clinicians [33].

Both the Headspace app and the web-based eMindful application provide family members with content on mindfulness. These applications include a virtual classroom offering a range of courses [34].

iFOCUS is designed as a psychoeducational programme that provides both general information on specific topics and personalised information tailored to the individual needs, priorities, and circumstances of family members and patients. Users receive a personalised workbook that can be downloaded after completion [25, 26].

Pep-Pal also adopts a psychoeducational approach by teaching family members about stress triggers, the effects of stress, and strategies for stress management. The evaluation showed a significant increase in participants’ ability to learn and apply stress management techniques. Overall, the session content was rated as helpful [31, 36].

In RLP, family members receive information on stress management techniques and on building resilience [32].

The CCP includes information on advance care planning and the use of supportive services. In the evaluation, the telephone follow-up was regarded as an informative source of support. Relatives valued the opportunity to engage with professionals [28, 29].

In the moderated Facebook support groups, knowledge is delivered through videos and supplemented by articles and linked websites. The content focuses on topics such as hospice care, pain management, and decision-making processes. This content has not yet been evaluated separately [35].

#### Mental and psychosocial support and enhancement of quality of life

Thirteen articles addressed the provision of mental and psychosocial support, as well as the quality of life of family members [23–29, 31–36].

The study by Bamgboje-Ayodele et al. [23] on the development of the Care Assist app found that male relatives of women with breast cancer often struggled psychologically with the situation, experienced self-doubt, and expressed a need for support in this regard. In addition, a need for peer support through the exchange of experiences was identified. The Delphi study by Goria et al. [24] yielded similar findings. The provision of emotional support and the development of skills to manage emotions and stress were regarded as particularly important.

The Pep-Pal intervention aims to explain the relationship between body and mind and to promote the development of techniques to strengthen mental resilience. In interviews, relatives reported that the app reminded them to attend to their own needs and to balance caregiving with other responsibilities. Furthermore, the evaluation suggested that Pep-Pal helped to reduce feelings of guilt, normalise emotional responses, and decrease feelings of isolation and loneliness [31, 36].

RLP focuses on strengthening resilience through a range of approaches. The evaluation showed significant improvements in stress levels and quality of life among family members. The programme also had an effect on stress, anxiety, and fatigue [32].

The web-based CHESS intervention allows family members to record their stress symptoms and offers psychosocial support through regular contact with clinicians. This approach strengthened collaboration between relatives and clinical professionals. According to the evaluation, early intervention when symptoms arise had a preventive effect on caregivers’ emotional burden [33].

The online diary ONDIARY is intended to enhance the emotional competence of family members. Daily entries are designed to reduce stress and promote mental well-being. The evaluation showed that the intervention group achieved significantly better outcomes than the control group in terms of quality of life, psychological distress, and daily life. However, no significant differences were found for depressive symptoms [27].

The Headspace and eMindful programmes significantly improved stress levels and mindfulness. According to the evaluation, family members used the apps specifically to reduce stress and to find ways of coping with anxiety [34].

The CCP focuses on promoting quality of life and addressing stress, frustration, grief, and depression. The evaluation showed that relatives in the intervention group improved significantly more than those in the control group. In qualitative interviews, the videos were described as particularly helpful. Participants were able to identify with the scenarios presented, gain a better understanding of their own emotions, and learn strategies to manage them. They also developed greater empathy for the patients. The emotional support provided through telephone contact was viewed positively [28, 29].

The moderated interaction within the Facebook groups was intended to provide emotional support through the sharing of stories. Members experiencing severe emotional or psychological distress were referred to staff at their local hospice. The evaluation did not identify any changes in quality of life or stress levels [35].

The iFOCUS components aimed at mental and psychosocial support addressed aspects such as outlook, resilience, meaning, coping, self-efficacy, and the reduction of uncertainty. The evaluation did not show any significant effects [25, 26].

#### Physical and practical support

The interventions addressing physical support and practical assistance vary in their objectives. Some primarily provide information about available support services, whereas others offer concrete guidance on actions that may help family members in everyday caregiving situations. Six interventions cover this area [23–31, 33, 36].

One of the Pep-Pal sessions focuses on how family members can access support. According to the evaluation, this helped participants to remember to balance caregiving responsibilities with other aspects of daily life [31, 36].

Within the CHESS application, notable entries are reported immediately to clinicians during check-in. Clinicians can then provide physical support, assist with symptom management, and help reduce emotional distress at an early stage [33].

As part of the CCP, family members receive information about available practical support resources. The programme also encourages them to seek practical help. The evaluation indicates that the practical advice and case examples were perceived as helpful for application in everyday life [28, 29].

The D-SCAN app includes signposting to support services [30].

The web-based iFOCUS programme includes references to external resources and contacts for practical support, such as self-help groups. It also provides suggestions intended to encourage users to take action, for example by introducing coping strategies and promoting a healthy lifestyle [25, 26].

The qualitative study by Bamgboje-Ayodele et al. [23] explored the requirements for a digital application for male caregivers of women with breast cancer, showing that practical support and concrete guidance are important. The development phase of Care Assist supports these findings. The study by Goria et al. [24] highlights the importance of providing instructions on dealing with emotions and stress, as well as guidance on caregiving tasks.

The Pep-Pal intervention also includes such guidance in the form of “Mini-Peps”, short video tutorials designed to promote relaxation, improve mood, and help maintain relationships [31, 36].

#### Communication support

Communication support is a component of five interventions [23–29, 31, 36].

The development phase of the web-based Care Assist intervention indicates that male caregivers would like support with effective communication. For instance, they want to know which questions are important to ask and how best to communicate with the affected person. They also expressed a need for a communication platform through which they could exchange experiences with others in similar situations [23, 24].

One Pep-Pal session focuses on managing relationships and communicating needs. The programme also seeks to improve communication about intimacy. However, the evaluation indicates that participants often rated this session as less helpful, as intimacy was not a priority in their current situation [31, 36].

The CCP aims to improve communication between patients and their caregivers. To this end, various methods are used to facilitate the sharing of memories and the expression of feelings. Follow-up telephone calls are conducted to assess whether the selected communication strategy was appropriate [28, 29].

ONDIARY addresses communication indirectly. Daily entries encourage reflection on behaviours, insights, and moods, which may promote discussion and improve the way these issues are handled. The long-term aim is to strengthen emotional competence, including the improved expression of feelings and emotions [27].

The iFOCUS programme guides family members in reflecting on their strengths, problems, and needs in relation to various topics. The intervention is completed jointly by patients and family members in order to promote exchange and foster mutual understanding [25, 26].

#### Preparation for caregiving and death

The online intervention Care Assist, which is currently in the development stage, is also intended to address preparation for caregiving and death. Male relatives of women with breast cancer, as well as experts, identified both dealing with caregiving tasks and preparing for the patient’s death as relevant topics. To support preparation for death, the creation of audiovisual material for later remembrance, such as voice messages and videos, was considered helpful [23, 24].

Preparation for caregiving situations or for the patient’s death is also addressed in two further technology-based interventions [28, 29, 35].

One module of the CCP is designed to help relatives of people with advanced cancer cope with frustration, depression and grief. This includes providing information about the stages of grief, helping participants to understand common triggers and causes of these emotions, and developing strategies to manage them. The follow-up call also addresses positive aspects of the caregiving situation. The evaluation indicates that this support was perceived positively [28, 29].

The moderated Facebook groups described by Parker Oliver et al. [35] are intended to support relatives of hospice patients with cancer by providing information about hospice care, assisting with decision-making, and offering support throughout the dying process. Group members can join or leave depending on their current situation. The evaluation showed significant improvements in anxiety and depression.

## Discussion

The analysis of the identified studies indicates that technology-based services specifically designed for informal caregivers of people with advanced cancer are available in several countries. However, the studies also show that different cancer types and disease stages are associated with differing needs, particularly with respect to information [17, 25, 29, 31, 32].

The interventions reviewed were often developed on the basis of preliminary studies or adapted from existing face-to-face programmes. Only a small number appear to be grounded in an explicit theoretical or evidence-based framework.

The interventions identified in this review focused primarily on mental and psychosocial support. Additional aims included the provision of information and resources, the communication of knowledge, and guidance on practical actions in caregiving and self-care contexts. Some programmes also addressed preparation for disease progression, caregiving situations, and death.

The evaluations of these interventions reported predominantly positive findings, particularly with regard to improvements in quality of life and reductions in depressive symptoms and anxiety. The support provided, flexible access to the applications and digital contact with specialists were also rated as helpful. Furthermore, the studies describe positive effects with regard to learning and applying coping strategies in stressful situations.

The findings of this scoping review highlight an important gap in the current landscape of digital health interventions. With regard to the underlying research question, the results indicate that relatives of patients with advanced breast cancer were sometimes included within broader target groups, but that no application specifically tailored to this population has yet been fully developed, implemented, and evaluated. The application currently under development, Care Assist, which is intended for male relatives of women with breast cancer, appears promising. However, no recent information is available on its current status, further development, or readiness for implementation. Tailored support services are likely to be needed for this target group, as informal caregivers are a highly heterogeneous population in terms of age [6, 7], and family caregiving in this context often involves men [23, 24].

The included studies also showed several methodological limitations. Small sample size was a particularly common issue [27, 29–33, 35], as was limited sample heterogeneity [23, 24, 29–33, 35, 36]. These limitations reduce both the generalizability of the findings and the strength of the conclusions that can be drawn. Additional limitations reported by the study authors included the lack of a specific evaluation of the application’s knowledge content [35] and the difficulty of assessing long-term effects [27].

### Study limitations

This scoping review has several limitations. Despite the comprehensive search strategy, relevant studies may have been missed because of the restriction to selected databases and to publications in English and German. In keeping with the nature of a scoping review, the methodological quality of the included studies was not formally assessed. In addition, commercially available applications that have not been evaluated in scientific studies could not be considered.

### Implications

The findings underline the need for future research to develop and evaluate digital interventions specifically tailored to informal caregivers of people with advanced breast cancer. They also suggest that such interventions should address heterogeneous support needs, provide flexible and low-threshold access, and facilitate contact with appropriate professional support where needed. These findings are also relevant to the project Caring Together for Advanced Breast Cancer (Gesi-BK), which aims to develop a digital support service for relatives and informal caregivers of people with advanced breast cancer. According to the current project concept, this service comprises (a) an information database with multimedia content, (b) digital personalised counselling, and (c) digital case management. The web-based application is intended to undergo both formative and summative evaluation. More broadly, the findings of this review suggest that future development in this field should place greater emphasis not only on intervention design and evaluation, but also on sustainable implementation within routine care.

### Conclusion

The results of this scoping review indicate that there are currently no technology-based applications specifically available for relatives of patients with advanced breast cancer. The review also highlights key features that may be important in the development of future support services, including responsiveness to diverse support needs, flexible and accessible delivery, and opportunities for contact with specialists. At the same time, the findings suggest that many digitally supported interventions do not progress beyond the project phase into sustainable long-term use. This points to persistent challenges in the implementation and maintenance of externally funded technology-based interventions. Sustainable implementation pathways, including viable funding or business models, should therefore be considered from the outset.

## Supplementary information

Below is the link to the electronic supplementary material.

ESM 1 (DOCX 108 KB)

ESM 2 (DOCX 30.2 KB)

## Data Availability

No new data were generated or analysed in this study. Data sharing is therefore not applicable.
