# Supplementary material for: Technology-based applications for relatives and caregivers of people with advanced breast cancer—a scoping review
Source: Support Care Cancer. 2026 May 5;34(6):501. doi: 10.1007/s00520-026-10712-z (PMC13144206; doi:10.1007/s00520-026-10712-z)
Supplement: Supplementary file 2 — (DOCX 30.2 KB) [file 520_2026_10712_MOESM2_ESM.docx]

**Appendix 2: Overview of the Included Studies**

| **Results** | - educational content: treatment options, side effects, caregiving, task  coordination, financial support  - psychological needs: coping mentally with the situation, support and  acceptance, relationship with healthcare professionals influencing  psychological well-being, preparation in case the cared-for person dies  - interactive and personalized intervention: online intervention via  internet/smartphone; includes not only text but also audio, graphics,  videos; tailored to individual needs  - interactivity through online group chat with men in same situation  - timing of intervention: starting at diagnosis, to accompany the process | - Pep-Pal is a Psychoeducation and Skills-based mobilized intervention  - nine sessions, focusing on stress, uncertainty and relationships  - Mini-Peps with video guided activities  - evaluation shows good acceptability and usability  - 4 major themes in interviews, related to the caregiver experience: putting the caregiver first, guilt, isolation and loneliness, latent traumatizing effects | - RLP is a psychosocial, mindfulness-based intervention (techniques for stress management, building resilience), 4 sessions  - program was appropriate to current life situation and easy to use/understand  - number and length of sessions acceptable  - caregivers showed improvements in distress, anxiety and fatigue and significant improvement in stress and quality of life  - practicing on a regular basis is important (gratitude, positive attitude, mindfulness)  - forgiveness was most challenging to implement | - no differences between groups regarding preparedness and burden, in  group with clinical report negative mood was lower (after 6 and 12  months)  - caregiver–clinician cooperation was stronger due to support  - clinicians can help manage uncontrolled symptoms and provide direct  support  - earlier intervention possible → prevention and less emotional stress for  caregivers because symptoms are under control |
| --- | --- | --- | --- | --- |
| **Methodology** | - qualitative, telephone interviews  - male relatives  - part of a mixed-methods study  - inductive analysis (Braun & Clarke)  - Australia  - n = 13 | - mixed-methods: quantitative & qualitative  - comparison of intervention group and control group  - USA   - - n = 56 | - mixed-methods: quantitative & qualitative  - one group, pre-post-design  - USA  - n = 33 patients and n = 15 caregivers | - inclusion of patients with advanced/metastatic  breast, prostate, lung cancer and their caregivers  - n = 235 patients and caregivers  - 42 % with breast cancer in the control group / 40 %  with breast cancer in the intervention group  - USA  - one group with CHESS use only, and one group  with CHESS use plus clinical report  - survey at 6 and 12 months |
| **Year** | 2020 | 2019 | 2025 | 2013 |
| **Title** | What male caregivers of women with breast cancer want in an online intervention: A qualitative study | Impact of a mobilized stress management program (Pep-Pal) for caregivers of oncology patients: mixed-methods study | Resilient living program for patients with advanced cancer and their caregivers | Communicating advanced cancer patients symptoms via the Internet: A pooled analysis of two randomized trials examining caregiver preparedness, physical burden and negative mood |
| **Authors** | Bamgboje-Ayodele A, Levesque JV, Gerges M, Girgis A | Carr AL, Jones J, Milkulich Gilbertson S, Laudenslager ML, Kutner JS, Kilbourn K, Sannes TS, Brewer BW, Kolva E, Joshi T, Pensak NA. | Chesak S, Rhudy L, Cutshall SM, Leventakos K, Tofthagen C, Mandrekar J, Rummans TA, Clark, MM, Ehlers S, Lapid MI, Sood A, Pachman DR | Chih M-Y, DuBenske LL, Hawkins RP, Brown RL, Dinauer SK, Cleary JF, Gustafson DH |
| **Results** | - iFOCUS is a self-managed online program, designed for autonomous completion, 4 sessions  - no statistically significant difference between the groups iFOCUS and standard care | Particularly important topics (consensus):  Basic information about breast cancer; types of treatment; factors influencing the decision to undergo treatment; expectations of treatment; side effects of treatment; life after treatment; dealing with emotions; coping with stress; sexual relationship with the patient; trust in healthcare; providing emotional support; guidance on how to be supportive; communicating with those affected; managing multiple caregiving tasks; fear of cancer recurrence | - significant effects for quality of life and psychological burden, financial  burden, and disruption of daily life (each with better outcomes in the  intervention group)  - no significant effects for depression | - mindfulness-based intervention via technology: “Headspace” or “eMindful”  - Caregivers showed significant  improvement in stress and mindfulness  after 6 weeks  - no significant difference  between intervention and control group  - reported motivations for using the  program: interest in mindfulness, coping  with anxiety, stress reduction; desire to  support patient  - benefits from mindfulness: living more in  the moment, learning to better cope  with stress and anxiety  - high satisfaction with the program |
| **Methodology** | - three-arm trial comparing FOCUS+ face-to-face intervention, iFOCUS web-based intervention and standard care  - quantitative, three time points  - two endpoints: emotional functioning, self-efficacy  - Europe  - n = 431 dyads (patients and caregivers) | - Delphi approach (online)  - rating the importance of individual items in two  rounds & providing comments  - expert survey  - n = 22  - Australia | - comparison of intervention and control group (n =  30 each)  - ONDIARY as an addition to standard care vs.  standard care  - relatives of breast cancer patients: 13 %  - inclusion of relatives of patients with advanced  cancer in home palliative care  - online questionnaire: quality of life and depression  - intervention group uses diary once per day for one  week  - Japan | - RCT, quantitative & qualitative  - inclusion of patients with advanced cancer and their  caregivers  - assessment of quality of life, stress, anxiety, depression,  and mindfulness  - majority with breast cancer: 31 % in the intervention  group & 49 % in the control group  - USA |
| **Year** | 2025 | 2022 | 2022 | 2020 |
| **Title** | Impact of a nurse-led and a web-based psychoeducational program for advanced cancer patients and their caregivers: Results of a three-arm randomized controlled trial | A Delphi-based approach to developing the contents of an online resource, Care Assist, for male caregivers of women with breast cancer | Effectiveness of the Online Daily Diary (ONDIARY) program on family caregivers of advanced cancer patients: A home-based palliative care trial | Pilot pragmatic randomized trial of mHealth mindfulness-based intervention for advanced cancer patients and their informal caregivers |
| **Athors** | De Vlemnick A, Matthys O, Turola E, Dierickx S, Dombrecht L, Van Goethem V, Deliens L, Lapeire L, Hudson P, Eecloo K, Brazil K, Groenvold M, Di Leo S, van der Heide A, Normand C, Harding R, Pilch M, Northouse L, DIAdIC Team, Cohen J | Goria S, Girgis A, Shaw J, Przezdziecki A, Levesque J, Bamgboje-Ayodele A | Ito E, Tadaka E | Kubo A, Kurtovich E, McGinnis M, Aghaee S, Altschuler A, Quesenberry C, Kolevska T, Liu R, Greyz-Yusupov N, Avins A |
|  | - CCP is a program for caregivers of a person with advanced cancer  - videos were helpful to understand emotions, understand patients and learn to cope  - telephone follow-up was a channel for emotional and informational support  - online forums were useful but they did not participate (barriers: time, not comfortable, no skills) | - CCP is based on Bandura’s self-efficacy theory  - significant improvements for all outcomes  - significantly higher improvements in intervention group  - CCP was well accepted and feasible | - Significant increase in caregivers’  psychological well-being  - raised awareness of available caregiving  support options | - educational content: information on hospice, pain management, decision making, dying process  - goal: safe online group for social support  - support aspects change as group membership and engagement change over time  - significant improvements in anxiety and depression  - no significant improvements in quality of life and burden |
|  | - qualitative, semi-structured individual interviews  - evaluation of caregivers perceptions  - Singapore  - n = 28 (n = 12 took part in interview) | - RCT, standard care + CCP vs. standard care  - quantitative, primary outcome: quality of life  - Singapore  - n = 80 (n = 42 in control group, n = 38 in intervention group) | - RCT  - Intervention group used an app, control group received standard care  - Standardized questionnaire survey  - n = 10 caregivers participated (all linked to patients who also participated, all in the intervention group)  - breast cancer: 42 % of patients (32 % in the intervention group and 52 % in the control group; largest proportion)  - UK | - clinical trial, three arms: control group, Facebook group, Facebook + web-conference group  - quantitative, outcomes: anxiety, depression, quality of life, burden  - USA  - n = 78 in Facebook group |
|  | 2016 | 2015 | 2022 | 2022 |
|  | Evaluation of a video, telephone follow-ups, and an online forum as components of a psychoeducational intervention for caregivers of persons with advanced cancer | A pilot randomized, controlled trial of the effectiveness of a psychoeducational intervention on family caregivers of patients with advanced cancer | A single-site pilot feasibility randomized trial of a supportive care mobile application intervention for patients with advanced cancer and caregivers | Facebook online support groups for hospice family caregivers of advanced cancer patients: protocol, facilitation skills, and promising outcomes |
|  | Leow M, Chan S | Leow M, Chan, S, Chan MF | Merz A, Mohamed A, Corbett C, Herring, K., Hildenbrand J, Locke SC, Patierno S, Troy J, Wolf S, Zafar SY, Chilcott J, Higgins A, Manassei H, McCoy C, Buckingham TL. LeBlanc TW | Parker Oliver D, Washington KT, Benson J, White P, Cruz Oliver D, Smith JB, Mazur J, Lakew A, Lewis A, Demiris G |
|  | - Pep-Pal: 12-week self-adminstered mobilized intervention to help caregivers of patients with advanced cancer manage stress, nine sessions  - additional brief “Mini-Peps”  - most utilized session: “Introduction to Stress Management”  - no significant difference between the groups regarding depression and anxiety  - intervention group: greater reductions for perceived stress, greater increases in ability to learn and use stress management skills and sexual functioning (women) | - Each component consists of four sessions  - All components are addressed in each  session  - Timeframe: 12 weeks, with 3 weeks  between each session  - Testing shows the program to be functional  and accepted  - Effectiveness is currently still being  evaluated |  |  |
|  | - RCT: Pep-Pal vs. standard care  - quantitative, outcomes: anxiety, depression, stress, health, sexual dysfunction  - USA  - n = 56 (n = 26 in Pep-Pal group, n = 30 in control group) | - iFOCUS developed based on FOCUS+  - Various perspectives included in the development  - 25 % breast cancer (largest share), 60 % stage IV cancer  (overall)  - Autonomous/independent use |  |  |
|  | 2020 | 2023 |  |  |
|  | A pilot study of mobilized intervention to help caregivers of oncology patients manage distress | A self-management psychoeducational eHealth program to support and empower people with advanced cancer and their family caregivers: Development using the scrum methodology |  |  |
|  | Pensak NA, Carr AL, Jones J, Mikulich-Gilbertson SK, Kutner JS, Kilbourn K, Sannes TS, Brewer BB, Kolva E, Joshi T, Laudenslager ML | Van Goethem V, Dierickx S, Matthys O, Northouse L, Lund L, Jordan C, Turola E, van der Wel M, Scott D, Harding R, Deliens L, Lapeire L, DIAdIC team, Hudson P, De Vleminck A, Cohen J |  |  |
